# Social Brain Functional Maturation in Newborn Infants With and Without a Family History of Autism Spectrum Disorder

**DOI:** 10.1001/jamanetworkopen.2019.1868

**Published:** 2019-04-05

**Authors:** Judit Ciarrusta, Jonathan O'Muircheartaigh, Ralica Dimitrova, Dafnis Batalle, Lucilio Cordero-Grande, Anthony Price, Emer Hughes, Johannes Klaus Steinweg, Johanna Kangas, Emily Perry, Ayesha Javed, Vladimira Stoencheva, Ranjit Akolekar, Suresh Victor, Joseph Hajnal, Declan Murphy, David Edwards, Tomoki Arichi, Grainne McAlonan

**Affiliations:** 1 Centre for the Developing Brain, School Biomedical Engineering & Imaging Sciences, King’s College London, St Thomas’ Hospital, London, United Kingdom; 2 Institute of Psychiatry, Psychology & Neuroscience, Department of Forensic and Neurodevelopmental Sciences, King’s College London, Denmark Hill, London, United Kingdom; 3 Sackler Institute for Translational Neurodevelopment, King’s College London, Denmark Hill, London, United Kingdom; 4 MRC Centre for Neurodevelopmental Disorders, King’s College London, London, United Kingdom; 5 Medway Hospital, Gillingham, Kent, United Kingdom; 6 South London and Maudsley NHS Foundation Trust, London, United Kingdom; 7 Department of Bioengineering, Imperial College London, London, United Kingdom

## Abstract

**Importance:**

What is inherited or acquired in neurodevelopmental conditions such as autism spectrum disorder (ASD) is not a fixed outcome, but instead is a vulnerability to a spectrum of traits, especially social difficulties. Identifying the biological mechanisms associated with vulnerability requires looking as early in life as possible, before the brain is shaped by postnatal mechanisms and/or the experiences of living with these traits. Animal studies suggest that susceptibility to neurodevelopmental disorders arises when genetic and/or environmental risks for these conditions alter patterns of synchronous brain activity in the perinatal period, but this has never been examined in human neonates.

**Objective:**

To assess whether alternation of functional maturation of social brain circuits is associated with a family history of ASD in newborns.

**Design, Setting, and Participants:**

In this cohort study of 36 neonates with and without a family history of ASD, neonates underwent magnetic resonance imaging at St Thomas Hospital in London, England, using a dedicated neonatal brain imaging system between June 23, 2015, and August 1, 2018. Neonates with a first-degree relative with ASD (R+) and therefore vulnerable to autistic traits and neonates without a family history (R−) were recruited for the study. Synchronous neural activity in brain regions linked to social function was compared.

**Main Outcomes and Measures:**

Regions responsible for social function were selected with reference to a published meta-analysis and the level of synchronous activity within each region was used as a measure of local functional connectivity in a regional homogeneity analysis. Group differences, controlling for sex, age at birth, age at scan, and group × age interactions, were examined.

**Results:**

The final data set consisted of 18 R+ infants (13 male; median [range] postmenstrual age at scan, 42.93 [40.00-44.86] weeks) and 18 R− infants (13 male; median [range] postmenstrual age at scan, 42.50 [39.29-44.58] weeks). Neonates who were R+ had significantly higher levels of synchronous activity in the right posterior fusiform (*t* = 2.48; *P* = .04) and left parietal cortices (*t* = 3.96; *P* = .04). In addition, there was a significant group × age interaction within the anterior segment of the left insula (*t* = 3.03; *P* = .04) and cingulate cortices (right anterior: *t* = 3.00; *P* = .03; left anterior: *t* = 2.81; *P* = .03; right posterior: *t* = 2.77; *P* = .03; left posterior: *t* = 2.55; *P* = .03). In R+ infants, levels of synchronous activity decreased over 39 to 45 weeks’ postmenstrual age, whereas synchronous activity levels increased in R− infants over the same period.

**Conclusions and Relevance:**

Synchronous activity is required during maturation of functionally connected networks. This study found that in newborn humans, having a first-degree relative with ASD was associated with higher levels of local functional connectivity and dysmaturation of interconnected regions responsible for processing higher-order social information.

## Key Points

**Question:**

Are changes in the maturation of the social brain in infants associated with vulnerability to neurodevelopmental conditions such as autism spectrum disorder?

**Findings:**

In this cohort study of 36 neonates with and without a family history of autism spectrum disorder, newborns with a family history of autism spectrum disorder had significantly higher neural activity in the right fusiform and left parietal cortex. In addition, the pattern of age-related changes in spontaneous activity in the cingulate and insula was disrupted in infants with a family history of autism spectrum disorder.

**Meaning:**

Atypical development of functional activity patterns in key regions responsible for social processing may be a vulnerability mechanism for autism.

## Teaser

This cohort study compares functional magnetic resonance imaging of the brains of neonates with a family history of autism spectrum disorder (ASD) vs those of neonates with no family history of ASD.

## Introduction

Social cognition refers to “the ability to perceive and process information from others, from one’s self and interpersonal knowledge.”^1^ The human brain has evolved to become more highly specialized in social interaction and communication than brains of any other species.^2^ This ability starts maturing early in life and quickly becomes highly complex through building on fundamental processes such as basic sensory integration and emotion recognition.^3^ Later in development, social function incorporates higher-order processes, such as mental state attribution, involving integrated activity in several regions across the whole brain. These regions make up the “social brain,” which has recently been defined by a meta-analysis of neuroimaging studies in social neuroscience as comprising 36 distinct regions. These can be further subgrouped into 4 main clusters: high-level subnetworks (involving attention, memory, and executive function), intermediate-level networks (responsible for cohesion of information in the social context), and visual-sensory and limbic networks (responsible for perception and integration of relevant cues).^4^

Anomalies in social interaction and communication are a core characteristic of autism spectrum disorder (ASD).^5^ It therefore comes as no surprise that abnormalities in functional connectivity within many of the regions composing the social brain have previously been reported in ASD. For example, ASD deficits in processing facial features have been associated with altered connectivity in the fusiform gyrus,^6,7^ difficulty with social demands has been associated with poor connectivity in the cingulate cortex,^8^ and aberrant processing of social stimuli has been associated with atypical activation patterns in the insula.^9^

However, the nature and extent of social brain anomalies associated with ASD and related conditions varies considerably. Moreover, the outcomes of those at risk of, or even diagnosed with, this neurodevelopmental condition are not fixed. This is likely a result of the multiple and complex primary and secondary compensatory mechanisms that occur throughout life. For example, well before difficulties become clinically evident at around 3 to 4 years of age,^10,11^ genetic expression is highly dynamic (with certain genes only expressing in particular developmental stages),^12^ and exposure to a wide range of potential protective (as well as harmful) environmental factors increases the heterogeneity of individuals with neurodevelopmental conditions.^13,14^ Also, we now know that parental interventions can modify the outcomes of infants at risk.^15,16^ Thus, what is inherited or acquired in autistic conditions is unlikely to be a specific disorder, but rather is a vulnerability to a spectrum of traits—and this may be modulated by a variety of factors throughout later development.^13^ Hence, to truly understand the biological mechanisms that confer vulnerability to ASD, we must look as early in life as possible.

Early-life studies of vulnerability mechanisms relevant to neurodevelopmental disorders have been largely conducted in animal models. Collectively, these reveal that synchronous activity in the perinatal brain is of fundamental importance as it is associated with local circuit connectivity and the development of functional networks.^17^ Disruption to these fundamental activity patterns is consistently reported in animals carrying either a gene mutation linked to ASD or exposed to prenatal environmental risk factors for ASD.^18,19,20^ Thus, based on the preclinical literature, early atypical synchronous activity may be a signature of vulnerability to neurodevelopmental conditions. However, this hypothesis has not been tested in humans, and specifically human neonates.

Although in the animal literature there are many types of synchronous activity described in the developing brain (eg, activity dependent on external experience or spontaneous intrinsic activity, characterized by different frequency oscillations and regulated by different receptors that rapidly change in early development^21,22^), in humans we are much more constrained by limited methods of data collection and analysis. Here we acquired resting state functional magnetic resonance imaging (fMRI) data in neonates during natural sleep. Our measure of synchronous activity was the Kendall correlation between blood oxygenation level dependent (BOLD) signal measured with resting state fMRI (natural sleep) at the individual voxel (volume unit) level and its nearest neighbors, namely, regional homogeneity (ReHo).^23^ Recognizing that infants with a family history of ASD have a higher likelihood of developing ASD traits than children without a family history, we defined vulnerable infants as those with a first-degree relative with ASD (R+). The R− infants had no family history of these conditions. We used ReHo analysis as a measure of local synchronous activity to compare regional differences in functional connectivity in the social brain. We predicted that synchronous activity patterns would be atypical in the social brain of vulnerable neonates.

## Methods

A total of 73 neonates were scanned at full term (>37 weeks) as part of the European Autism Interventions (EU-AIMS) Brain Imaging in Babies (BIBs) study approved by the South London National Research Ethics Committee from 2014 to the present. Written informed consent to participate in the study was taken from the parents of all participants prior to data acquisition. The study was conducted in accordance with the Strengthening the Reporting of Observational Studies in Epidemiology (STROBE) reporting guideline for cohort studies. At recruitment, participants were first carefully screened by experienced researchers to determine whether parents reported a family history of ASD. Next, at the time of the scan, a pediatric research nurse with a comprehensive understanding of the UK National Health Service diagnostic provisions for neurodevelopmental conditions interviewed the parent(s). This included taking an extended medical and psychiatric family history and informing each participant’s general practitioner of their participation in the study. A neonate was characterized as at risk if his or her parent provided a clear account that a parent and/or an older sibling had been diagnosed by the appropriate clinical services (namely, the UK Child Development Centres, Child and Adolescent Mental Health Services, or Adult Autism Assessment Services). The preparation of the neonate for scanning and monitoring in the scan took priority on the day and considerable time was spent explaining the protocol and ensuring the parents were well informed about the process. Therefore, if any details of the family risk history were not captured on the scanning day, those parents were also contacted again after the scan.

Twenty-nine families had either a previous child or a parent diagnosed with ASD, and 44 had no previous clinical conditions in their family history. After applying strict criteria to exclude fMRI data corrupted by head motion (due to high framewise displacement; see Data Processing subsection), results from 11 neonates were discarded from the original 29 in the R+ group and 11 were discarded from the original 44 in the R− group. This resulted in a data set consisting of 51 participants. Of these, 18 infants had a first-degree relative with ASD (and were therefore considered at risk of adverse neurodevelopmental outcomes) and were assigned to the R+ group; the remaining 33 composed the R− group. To control for an imbalance in sex (the R+ group had a higher ratio of male infants) 15 female infants from the R− group were randomly excluded. The final study group, therefore, consisted of 18 R+ and 18 R− participants (the characteristics of the sample are described in detail in Table 1). In the R+ group, 15 infants had a first-degree relative with ASD and 3 had a first-degree relative with both ASD and attention-deficit/hyperactivity disorder. In the R− group, there was no significant family history of neurodevelopmental conditions or major mental illness. Mann-Whitney *U* tests revealed no significant differences between groups in gestational age at birth, postmenstrual age at scan, weight at birth, and head circumference at scan. The gender distribution between groups was identical (detailed information of each participant is described in Table 2). Infants had no evidence of structural malformation on structural MR images (as reported by a neonatal neuroradiologist).

**Table 1.** Study Sample Descriptive Statistics

Demographic Characteristics	Median (Range)		*P* Value
	Risk for ASD (n = 18)	No Risk for ASD (n = 18)	
Birth gestational age, wk	39.79 (34.43-41.14)	39.57 (36.57-42.00)	.54
Scan postmenstrual age, wk	42.93 (40.00-44.86)	42.50 (39.29-44.58)	.36
Scan postnatal age, d	23.5 (1-52)	17.5 (9-45)	.12
Birth weight, kg	3.44 (2.80-4.70)	3.27 (2.52-4.25)	.73
Male, No./female, No.	13/5	13/5	NA
Head circumference, cm	36.15 (33-38)	35.40 (34-38)	.69

Abbreviations: ASD, autism spectrum disorder; NA, not applicable.

**Table 2.** Study Sample Participant-Specific Demographic Characteristics^a^

Patient No.	Sex	Gestational Age, wk	Postmenstrual Age, wk	Postnatal Age, d	Birth Weight, kg	Head Circumference, cm	Family Member Affected	Diagnosis
01	Male	40.57	43.86	23	3.37	37.9	Brother	ASD
02	Female	40.14	44.29	29	3.95	36.7	Brother	ASD
03	Male	35.43	42.86	52	3.06	33.0	Brother	ASD
04	Male	39.57	43.00	24	3.60	37.0	Half brother	ASD
05	Female	40.57	44.86	30	4.70	38.0	Sister	ASD
06	Female	39.29	40.57	9	2.94	34.0	Brother	ASD
07	Male	35.57	40.00	31	2.82	38.0	Brother	ASD
08	Male	34.43	41.43	49	2.80	35.0	Brother	ASD
09	Male	39.14	44.00	34	3.50	36.5	Brother	ASD
10	Female	41.14	44.00	20	3.10	34.0	Half brother	ASD
11	Male	39.00	41.14	15	2.84	35.0	Sister	ASD
							Brother	ASD
12	Male	40.29	42.71	17	3.90	37.5	Brother	ASD
13	Male	38.86	43.29	31	3.43	38.0	Brother	ASD
14	Male	40.71	40.86	1	3.84	34.0	Sister	ASD and epilepsy
15	Male	40.00	42.00	14	3.69	34.5	Brother	ASD
							Mother	ASD and ADD
16	Female	40.43	44.14	26	2.80	35.8	Father	ASD
17	Male	39.00	42.00	21	4.10	38.0	Brother	ASD and dyspraxia
18	Male	40.43	43.29	20	3.44	35.2	Sister	ASD
19	Male	40.71	43.29	18	3.20	37.4	NA	No NDD
20	Male	39.14	41.57	17	3.15	34.3	NA	No NDD
21	Male	40.14	43.86	26	3.92	37.5	NA	No NDD
22	Male	39.14	41.71	18	3.29	37.5	NA	No NDD
23	Male	40.14	43.14	21	3.80	35.0	NA	No NDD
24	Male	37.86	40.29	17	3.34	34.0	NA	No NDD
25	Male	37.43	39.29	13	3.06	35.0	NA	No NDD
26	Female	38.57	41.14	18	3.25	35.6	NA	No NDD
27	Male	41.00	43.00	14	3.10	37.0	NA	No NDD
28	Female	40.57	42.86	16	3.02	34.4	NA	No NDD
29	Male	40.14	41.43	9	3.11	35.1	NA	No NDD
30	Female	38.86	41.29	17	4.25	36.5	NA	No NDD
31	Male	41.14	43.57	17	4.04	38.0	NA	No NDD
32	Male	36.57	43.00	45	2.52	35.2	NA	No NDD
33	Female	38.71	42.14	24	3.01	35.0	NA	No NDD
34	Male	38.86	40.57	12	3.60	35.0	NA	No NDD
35	Male	42.00	44.57	18	3.78	36.0	NA	No NDD
36	Female	40.00	43.57	25	3.44	36.0	NA	No NDD

Abbreviations: ADD, attention-deficit disorder; ASD, autism spectrum disorder; NDD, neurodevelopmental disorders.

^a^ The first 18 rows represent data for the participants who had a familial risk for ASD and the last 18 rows represent newborn infants with no family history of ASD or other NDD.

### Data Acquisition

Infants were studied during natural sleep following feeding and immobilization in a vacuum evacuated bag (Med-Vac; CFI Medical Solutions). Hearing protection (molded dental putty in the external auditory meatus [President Putty; Coltene Whaledent] and earmuffs [MiniMuffs; Natus Medical Inc]) and physiological monitoring (oxygen saturations, heart rate, and axillary temperature) were applied before data acquisition. Data acquisition sessions were attended by a clinician (pediatric physician or nurse) trained in neonatal resuscitation. While the newborn was in the scanner, the parents were in a waiting room with a video link that allowed them to view their baby at all times. High–temporal resolution resting-state fMRI data were acquired in a 3T Philips Achieva scanner located on the neonatal intensive care unit at St Thomas’ Hospital London with a customized 32-channel neonatal head coil and imaging system.^24^ We acquired BOLD fMRI data using a multislice gradient-echo echo planar imaging sequence with multiband excitation (multiband factor 9) with the following parameters: repetition time, 392 milliseconds; echo time, 38 milliseconds; spatial resolution, 2.15 mm isotropic; flip angle, 34°; 45 slices; total time, 15 minutes 3 seconds (2300 volumes). High-resolution anatomical T1-weighted images (spatial resolution, 0.8 mm isotropic; field of view, 145 × 122 × 100 mm; and repetition time, 4795 milliseconds) and T2-weighted images (spatial resolution, 0.8 mm isotropic; field of view, 145 × 145 × 108 mm; and parameters of repetition time, 12 000 milliseconds and echo time, 156 milliseconds) were acquired for registration and clinical reporting purposes.^24^ Further details about the imaging protocol, which takes approximately 1 hour, can be found in the article by Hughes et al.^24^

### Data Processing

Data preprocessing was carried out using an in-house pipeline specifically developed and optimized for sources of artifact inherent to neonatal fMRI data, including uncontrolled head motion and developmental differences in brain configuration. As head motion during fMRI data acquisition has been shown to significantly alter analysis and can lead to spurious patterns of connectivity,^25,26^ the acquired data were then cropped to 1600 continuous volumes with the least amount of motion (estimated by calculating the framewise displacement of each volume) for further analysis. Participants with high framewise displacement (>0.5 mm) in more than 80 volumes of the 1600 total volumes (5% of the cropped data set) were excluded entirely from analysis. Independent component analysis was then used to identify signal artifacts (those related to residual head motion, multiband acquisition, and physiological fluctuations, eg, cardiac pulsation and respiratory motion) and to train a classification algorithm for automatic identification of these sources of artifact, which were then regressed out from the data using the FSL FIX tool.^27,28^ Next, we used the FSL topup tool to estimate the warp embedding field distortions caused by susceptibility artifacts.^29^ We use this warp, combined with each participant’s anatomical T2, to nonlinearly transform each participant’s functional data to a 41-week-old neonatal template.^30,31^

### Statistical Analysis

**Figure 1.**
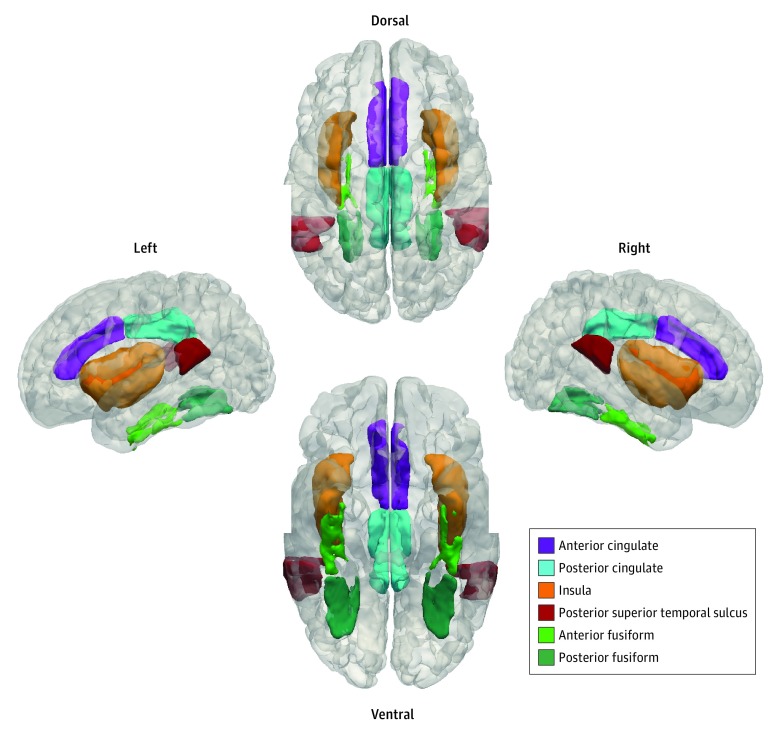
Anatomical Segmentation of Regions of Interest Relevant for Social Function The anterior cingulate, the posterior cingulate, the insula, the posterior superior temporal sulcus, the anterior fusiform, and the posterior fusiform are represented inside a 3-dimensional rendering of the neonatal template. Right, left, ventral, and dorsal views were selected to provide full visualization of the anatomical space covered by the regions of interest.

Voxelwise regional homogeneity was assessed by calculating the Kendall coefficient of concordance between the BOLD contrast time series of a given voxel with its 26 adjacent neighbors using the Analysis of Functional Neuroimages (AFNI) 3dReHo tool.^23,32^ Voxelwise ReHo Kendall τ was converted into Pearson *r* (*r* = sin[πτ/2]) and then transformed into Fisher *z* scores prior to statistical comparison.^33^ A neonatal labeled segmentation atlas^34,35^ was used to select the regions of interest (ROIs) pertinent to social function. The social brain ROIs were selected based on the recent meta-analysis that reported the social brain to be composed of 36 regions.^4^ Owing to immature anatomy and unclear boundaries in the neonatal brain, we restricted our study to 16 ROIs that could be clearly delineated on the neonatal atlas. These ROIs encompassed the anterior and posterior fusiform gyri, the anterior and posterior cingulate cortices, the posterior superior temporal sulcus, the insula cortex (Figure 1), the parietal cortex, and the frontal cortex, all bilaterally. The anterior temporal lobe was selected as a control region, as it is thought to rely on the use of abstract concepts in social cognitive processes, which are still very immature at this stage of human development.

Group differences in ReHo *z* scores were tested within each ROI using permutation testing as implemented in FSL’s Randomize (v2.1)^36^ with false discovery rate correction for multiple comparisons (significance threshold set to *P* < .05 based on 2-tailed analysis). The general linear model was controlled for gestational age at birth, postmenstrual age at scan, and sex. A separate general linear model was used to test the hypothesis that there would be group × age–related differences in ReHo within the social brain network of interest. This model was controlled for sex (false discovery rate–corrected significance threshold, *P* < .05). Finally, both models were used for a whole-brain exploratory analysis to determine whether any between-group or age-related differences observed within the social ROI analyses could be generalized across the rest of the brain (false discovery rate–corrected significance threshold, *P* < .05).

## Results

### Main Effect on Synchronous Activity

**Figure 2.**
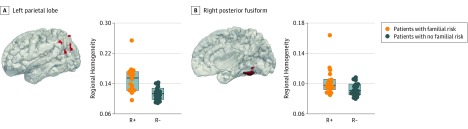
Higher Levels of Regional Homogeneity in Several Clusters in the Social Brain Among Neonates With Familial Risk for Autism Spectrum Disorder (R+) Compared With Neonates With No Family History (R−) The 3-dimensional rendered clusters (red) where there was a significant main effect of group (*P* < .05 after false discovery rate correction) are overlaid on a neonatal template. These clusters were localized to the left parietal lobe (A) and the right posterior fusiform (B). The filled circles in the plots represent the median regional homogeneity value per participant of all the voxels located in the significantly different clusters; boxes represent the quantiles; and the central horizontal line, the median.

The final data set consisted of 18 R+ infants (13 male; median [range] postmenstrual age at scan, 42.93 [40.00-44.86] weeks) and 18 R− infants (13 male; median [range] postmenstrual age at scan, 42.50 [39.29-44.58] weeks). Compared with the R− group, infants in the R+ group had higher ReHo levels in the right posterior fusiform (*t* = 2.48; *P* = .04) and the left parietal cortex (*t* = 3.96; *P* = .04) (Figure 2). This included the fusiform face area in the right hemisphere (Figure 2). There were no other significant differences in average ReHo values (either higher or lower) between groups in any of the other social ROIs studied. There were also no significant differences in average ReHo in the control regions (anterior temporal lobes) or across the whole brain between groups (eFigure in the Supplement).

### Age Interaction Effect

**Figure 3.**
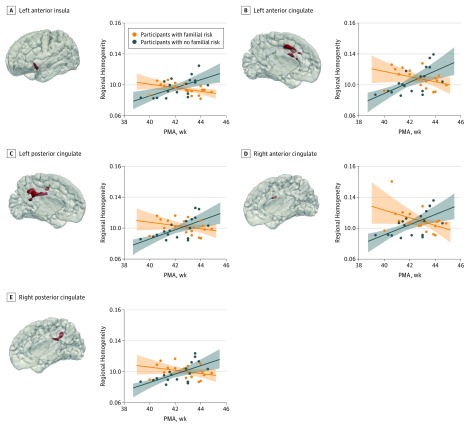
Significantly Different Maturational Trajectory in Cingulate and Insula Among Participants With Familial Risk of Autism Spectrum Disorder The 3-dimensional rendered clusters overlaid in a neonatal template (red) show the voxels that appeared to have a significantly different age interaction effect between groups. The clusters were located in left anterior insula (A), left anterior cingulate (B), left posterior cingulate (C), right anterior cingulate (D), and right posterior cingulate (E). For each region of interest, we plotted the median regional homogeneity value (lines) with 95% confidence intervals (shaded area) and show how these values increase with postmenstrual age (PMA) for the group with no familial risk, while they remain constant or decrease for the group with familial risk.

A separate voxelwise test of the interaction between groups and the linear effect of postmenstrual age on ReHo values was performed for each of the social brain ROIs. Significant group × age interactions were identified in the left insula (*t* = 3.03; *P* = .04), right (*t* = 3.00; *P* = .03) and left (*t* = 2.81; *P* = .03) anterior cingulate, and right (*t* = 2.77; *P* = .03) and left (*t* = 2.55; *P* = .03) posterior cingulate cortices (Figure 3), with R+ participants having higher ReHo levels compared with R− neonates at a younger postmenstrual age, while those scanned when older showed the reverse. Within these regions, there was a clear maturational pattern of increasing ReHo values over 39 to 45 weeks’ postmenstrual age in the R− group in comparison with a slight decrease (or minimal change) in R+ infants. This same trend was seen in each group for all other social brain regions, although it did not reach statistical significance. In contrast, there was no group × age interaction in ReHo either across the whole brain or within the preselected anterior temporal lobe control regions (eFigure in the Supplement).

## Discussion

In this study, we show that by the normal time of birth, regions involved in social cognition had already begun to mature differently in neonates vulnerable to autistic conditions. We report higher ReHo levels localized to the posterior right fusiform gyrus (including the face-specialized region in at-risk neonates) and left parietal cortex. We also report significantly different age-related cross-sectional changes within the bilateral cingulate and the insular cortices. These findings in R+ infants were specific to the social brain; we observed no group differences in ReHo in a predefined control region (anterior temporal lobe), nor any global or maturational differences in local functional connectivity across the whole brain.

### Group Differences in the Social Brain

Located on the ventral surface of the temporal cortex, the fusiform gyrus is a key structure in high-level visual functions such as face recognition, color processing, and word recognition.^37,38,39^ The structure is distinct to hominoids and is likely to be one of the earliest areas involved in social cognitive tasks to mature according to the interactive specialization theory. This theory postulates connectivity and activity patterns in early maturing networks, such as the fusiform,^40^ will interact with other regions to ensure later maturing networks acquire the intended social cognitive function.^41^ Among the 4 social clusters, the fusiform is in the visual sensory cluster, which is responsive to biologically relevant cues related to perception-action processes in social cognition and essential for a person to interact with her or his environment.^4^ Despite their relatively limited visual capacities, neonates have strong preferences toward facelike stimuli. They even demonstrate characteristics of mature face recognition abilities, such as an ability to discriminate between different emotional expressions.^42,43,44^ Differences in face processing emerge in infancy in ASD, as evidenced by a recent study reporting different attention levels in R+ newborn infants compared with an R− cohort.^45^ This is consistent with our finding that there are already differences between the R+ and R− groups that involve the fusiform face area, particularly in the right posterior fusiform, which is known to be associated with face processing.^46,47^ Thus, increased synchronous activity in the social brain and fusiform face area, specifically, may constitute a vulnerability marker for social traits in ASD, and future studies might benefit from combining this type of analysis with behavioral measures of attention to facelike stimuli.

In addition to differences in the fusiform gyrus, we also observed significant differences between groups in the left parietal lobe. The left parietal cortex is part of the default mode network, showing strong functional connectivity with the frontal cortex, and has been associated with self and social information retrieval.^48^ The involvement of this part of the default mode network in our findings fits with evidence that the posterior regions of this network mature prior to frontal cortices.^49^ Thus, our observations may indicate atypical maturation of a network associated with social priming during rest; this is consistent with recent studies in adolescents with ASD that have found an association between underconnectivity in this area and atypical self-other processing abilities.^8^

### Group Age-Associated Differences in Early Development

Face-processing difficulties are thought to be a precursor of the alterations in higher-order processing, such as joint attention, that develop later in infancy.^50^ Joint attention skills develop in the first 6 months following birth and require the establishment of a common frame of reference between the infant and the adult in order to share information about an object or an event. The primary nodes of the mature joint attention system include the frontal cortex, the insula, the cingulate, the superior temporal cortex, the precuneus, and the amygdala.^51^ Of these regions, we were constrained by the neonatal atlas used for this study and could only investigate the insula, the cingulate, and part of the superior temporal cortex. These regions are all components of the intermediate level cluster in the social cognition atlas, with the anterior cingulate also being involved in the limbic cluster.^4^ The interaction effect analysis found that R+ neonates show different age-associated changes in the insula and cingulate. While synchronous activity appears to be increasing with age in the R− group, a change with increasing age is not evident in R+ infants.

Increasing functional connectivity with age at this early phase of development is in agreement with previously reported results of functional abnormalities in the insula in the neurodevelopmental spectrum. The insula has been found to be an important functional hub region in the infant brain, with significantly increases in mean functional connectivity demonstrated in neonates to 1 year, but not during the second year in typical development.^52^ Interestingly, studies in older children with ASD have reported both greater activity in the insula when performing an attention task^9^ and lower activity in the insula when tested for theory of mind.^53^ This variability also extends to other regions such as the cingulate, within which lower activity has been reported during tasks involving theory of mind,^53^ while others have reported higher intranetwork cingulate connectivity at rest.^54^ These discrepancies in results from investigations of older populations may reflect different compensatory activity patterns during task and rest situations, or an aberrant activity pattern that accompanies different maturational profiles in this heterogeneous spectrum. The challenges interpreting data from older cohorts underscore the need to minimize confounding factors and examine vulnerability mechanisms in early development. Until recently, we have had to depend on animal studies to examine early development. While these have successfully provided insight,^18,19^ they fall short of replicating the complex social brain of humans. Most notably, rodents do not have a specialized face-processing region in the brain, so work in human infants is essential.^2^

### Absence of Differences in Regions Involved in Higher-Order Processes

The anterior temporal lobe is considered critical for merging the multicategorical information required to process very abstract social representations of another person.^55,56,57^ However, this form of abstract higher-order processing is likely relatively underdeveloped in a newborn infant, which therefore made the region a suitable control area for our purposes. We observed no differences between groups in these regions with any of the models tested, further suggesting that vulnerability to atypical development is time dependent and differs among regions involved in social cognition that mature at different developmental points.

### A Vulnerability Marker for Autistic Traits

The outcomes of individuals at risk of, or even diagnosed with, ASD are highly diverse.^14^ Therefore, a vulnerability marker for ASD should ideally reflect a mechanism that allows for heterogeneity of outcomes (ie, it should be modifiable, for example, by postnatal experience). The higher levels of ReHo observed here may meet this requirement. In animal systems, under the influence of the external postnatal environment, spontaneous activity typically becomes sparse once a region is mature but can follow different trajectories depending on genes and environment.^58^ We suggest that parallels can be drawn with the work carried out in infants here. Thus, we propose that the abnormal prolongation of synchronous neural activity in R+ infants is a potential marker of immaturity, and multifactorial influences in postnatal life will subsequently determine the rate and extent to which a mature pattern of activity is achieved. What causes higher ReHo in the R+ at birth is, however, difficult to know. One possibility is that it is a response to disruption within a prenatal sensitive period^59^; whether this is a primary pathological or a secondary compensatory mechanism needs to be investigated further in both preclinical and clinical work that not only follows up children at risk but also examines precursors in the prenatal period.

### Limitations

Our dedicated neonatal MR imaging system,^24^ optimized high–temporal resolution fMRI, and data analysis pipeline allowed us to mitigate specific challenges associated with neonatal fMRI studies. For example, the high spatial resolution of our fMRI data (2.15 mm isotropic) and the spatial normalization of our data to an age-specific structural atlas^30^ limited partial volume errors and helped constrain analysis to the cortex.^60^ Because head motion can lead to signal artifact and affect estimates of functional connectivity,^61^ we applied strict data exclusion criteria for head motion, analyzing only 1600 continuous volumes of the total 2300 acquired in each data set to ensure that absolute head displacement was less than 0.5 mm for 95% of the data and discarding data sets entirely from infants in whom greater than 5% of the acquired volumes were corrupted by a framewise displacement of more than 0.5 mm. Although this reduced the total duration of the available time series for each infant to 10 minutes 27 seconds, the very high temporal resolution of the data ensured that our ReHo measurements remained robust.^62^ This high sampling rate also allowed improved removal of physiological noise, which can be problematic in infants with naturally higher breathing and cardiac pulsation rates.^63^ It is also known that sleep state can alter functional connectivity measures and may represent a potential confounding factor in our study. However, neonatal sleep consists of only 2 stages (active and quiet),^64^ and it is currently unknown whether connectivity measures differ during these early sleep states in a way similar to how they do in the mature brain. In addition, given that head motion may be a proxy for the level of arousal,^65^ because we discarded data with large degrees of head motion, our population was likely to be predominately in quiet sleep during study. Nonetheless, we aim to better characterize the effect of physiological parameters and sleep in future studies.

Another limitation, or rather a novelty, in our study is the inclusion of a parent with ASD as a risk for ASD in the offspring. This is based on the assumption that for a child with ASD (the sibling) there was likely a causal contribution of one or both parental genetic risk. Thus, it is reasonable to consider the parents as conferring risk to their offspring. This is supported by evidence that the parents of children with ASD have high levels of autistic traits.^66,67^ In addition, many families who go on to have a child with ASD remain singleton families, and these have been relatively neglected in the at-risk design. By including children without a sibling in the at-risk group we hope to be more representative of those families. We also elected to include neonates with a half sibling with ASD and to assign these children to the R+ group. We would expect that if these children do have a lower risk of ASD than the rest of the group, they would lessen the chance of us observing significant differences on the measures analyzed. A further limitation is assigning risk status based on family history, which excludes infants who might have a de novo predisposition to ASD. It is now appreciated that risk for ASD is multifactorial and includes, for example, prenatal exposure to maternal depression and/or antidepressant medication, inflammation, and birth complications. Thus, future studies will be needed to examine whether a dysmaturation within the social brain can be generalized to these other risk groups. The interpretation of our findings may be potentially limited by the small sample size, which may have led to false negative findings as a consequence of low power. To mitigate this, we used nonparametric methods throughout our analyses to avoid bias through making assumptions about the data distribution. However, it will be crucial to replicate the study in a larger data set. We also acknowledge that a truly longitudinal design would be helpful, but for practical purposes having very young infants attend repeatedly even over the first weeks of life is challenging, let alone within the short periods over which the brain changes so dynamically in infancy. We acknowledge the relatively narrow age range of our study. However, we view this as a strength, as during this period of infancy the child develops incredibly rapidly. For example, the sensitivity to contrast in visual stimuli rapidly changes after birth.^68^

## Conclusions

We found that newborns with vulnerability to ASD showed significant differences in synchronous activity levels and maturation within core components of the social brain. This implies that a deviation from the typical maturational trajectory of the social brain circuits must be emergent in utero. Future fetal and neonatal imaging studies will help map the development of the social brain and could usefully examine what (diet, stress, or inflammation) might modify activity in the typical infant or even what might normalize aberrant activity in children who are vulnerable to adverse outcomes.

## Notes

Supplement. **eFigure.** No Significant Differences in Anterior Temporal Control Region and Whole Brain
