# Supplementary material for: Social Brain Functional Maturation in Newborn Infants With and Without a Family History of Autism Spectrum Disorder
Source: JAMA Netw Open. 2019 Apr 5;2(4):e191868. doi: 10.1001/jamanetworkopen.2019.1868 (PMC6450332; doi:10.1001/jamanetworkopen.2019.1868)
Supplement: Supplement. — eFigure. No Significant Differences in Anterior Temporal Control Region and Whole Brain [file jamanetwopen-2-e191868-s001.pdf]

Supplementary Online Content

Ciarrusta J, O'Muircheartaigh J, Dimitrova R, et al. Social brain functional maturation in newborn infants with and without a family history of autism spectrum disorder. *JAMA Netw Open*. 2019;2(4):e191868. doi:10.1001/jamanetworkopen.2019.1868

eFigure. No Significant Differences in Anterior Temporal Control Region and Whole Brain

This supplementary material has been provided by the authors to give readers additional information about their work.

© 2019 Ciarrusta J et al. *JAMA Network Open.*

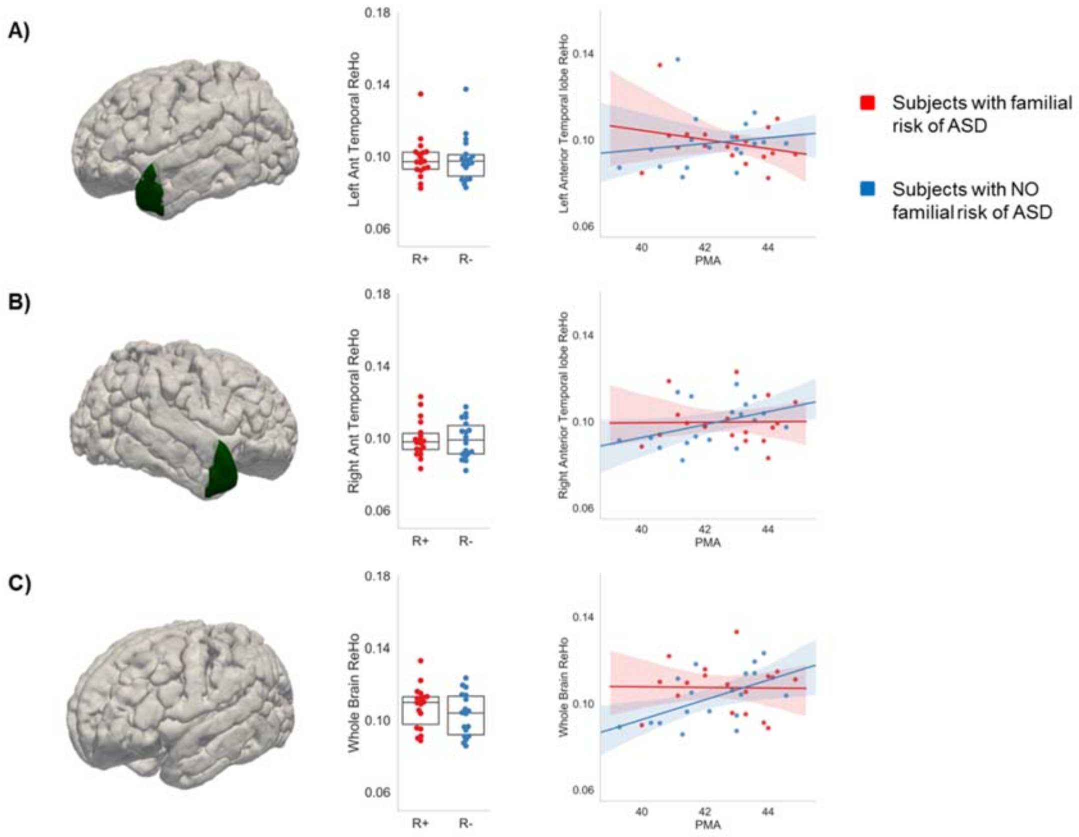

eFigure. No significant differences in anterior temporal control region and whole brain. The 3D rendered masks overlaid in a neonatal template (green) show the temporal regions that have been associated to processing of higher order abstract social concepts in the adult brain (A-B). For each control region (A-B) and the whole brain (C) we plot the quantile and median values per group and the group age interaction with 95% confidence intervals to show we found no significant differences. (*Abbreviations: Autism Spectrum Disorder (ASD), with familial risk for ASD (R+), without familial risk for ASD (R-) Regional Homogeneity (ReHo), Postmenstrual Age (PMA), Anterior (Ant)).

© 2019 Ciarrusta J et al. *JAMA Network Open.*
